# Precision and False Perceptual Inference

**DOI:** 10.3389/fnint.2018.00039

**Published:** 2018-09-20

**Authors:** Thomas Parr, David A. Benrimoh, Peter Vincent, Karl J. Friston

**Affiliations:** Institute of Neurology, Wellcome Trust Centre for Neuroimaging, University College London, London, United Kingdom

**Keywords:** active inference, saccades, visual system, synucleinopathy, precision

## Abstract

Accurate perceptual inference fundamentally depends upon accurate beliefs about the reliability of sensory data. In this paper, we describe a Bayes optimal and biologically plausible scheme that refines these beliefs through a gradient descent on variational free energy. To illustrate this, we simulate belief updating during visual foraging and show that changes in estimated sensory precision (i.e., confidence in visual data) are highly sensitive to prior beliefs about the contents of a visual scene. In brief, confident prior beliefs induce an increase in estimated precision when consistent with sensory evidence, but a decrease when they conflict. Prior beliefs held with low confidence are rapidly updated to posterior beliefs, determined by sensory data. These induce much smaller changes in beliefs about sensory precision. We argue that pathologies of scene construction may be due to abnormal priors, and show that these can induce a reduction in estimated sensory precision. Having previously associated this precision with cholinergic signaling, we note that several neurodegenerative conditions are associated with visual disturbances and cholinergic deficits; notably, the synucleinopathies. On relating the message passing in our model to the functional anatomy of the ventral visual stream, we find that simulated neuronal loss in temporal lobe regions induces confident, inaccurate, empirical prior beliefs at lower levels in the visual hierarchy. This provides a plausible, if speculative, computational mechanism for the loss of cholinergic signaling and the visual disturbances associated with temporal lobe Lewy body pathology. This may be seen as an illustration of the sorts of hypotheses that may be expressed within this computational framework.

## Introduction

The brain's visual system must overcome formidable inferential challenges. Despite receiving sequentially sampled, spatially limited sensory information from a two-dimensional array of photoreceptors, we perceive spatially and temporally continuous three-dimensional visual scenes, populated with complex objects. However, despite this remarkable capacity for scene construction (Mirza et al., 2016; Parr and Friston, 2017a), the visual system is not infallible. It depends upon a delicate balance between prior beliefs about perceptual hypotheses (Gregory, 1980), and the sensory evidence that supports or refutes them (Geisler and Kersten, 2002; Brown and Friston, 2012). This paper is about the computational mechanisms that could maintain this balance, and the consequences of their failure (e.g., Collerton et al., 2005).

We begin by outlining active inference (Friston et al., 2015), a theory of optimal behavior that accommodates both perceptual inference and foraging for new sensory data (Mirza et al., 2016). For our purposes, this foraging takes place through saccades to locations in the visual scene. We simulate visual foraging and the concurrent optimisation of beliefs about the precision of (i.e., confidence in) sensations, using a simple visual scene. This serves to illustrate the sensitivity of perceptual inference to the prior beliefs chosen, and the potential for (pathological) false inference (Friston, 2017).

These considerations serve as a foundation for the next section, which explores the source of abnormal prior beliefs. To do so, we extend the model and relate inferential message passing to the interactions between the ventral visual stream and subcortical structures. This draws upon previous suggestions that acetylcholine may act as a sensory precision signal (Yu and Dayan, 2005; Parr and Friston, 2017c). This rests upon several empirical observations. First, nicotinic acetylcholine receptors are found on the presynaptic terminals of cells in layers 3 and 4 of the cortex (Sahin et al., 1992; Lavine et al., 1997). These laminae are the targets of sensory relays from the thalamus (Shipp, 2007). Secondly, cholinergic manipulations modulate the gain of visually evoked responses (Gil et al., 1997; Disney et al., 2007). This renders it almost tautologically true that one of the roles of acetylcholine is to modulate the precision of (some types of) sensory input, as precision and gain are mathematically identical. Finally, in both behavioral (Marshall et al., 2016) and neuroimaging (Moran et al., 2013) studies in humans that explicitly test the association between precision and acetylcholine, differences following cholinergic manipulations are best accounted for by altered precision. If we lesion our model to simulate the Lewy body pathology associated with visual disturbances in synuclein disorders (Harding et al., 2002), we find that the downstream consequences include decreased sensory precision, neuronal gain, and false perceptual inferences. These are consistent with the cholinergic deficits, occipital hypometabolism, and visual hallucinations that occur with synucleinopathies (McKeith et al., 2004). While this is speculative, we draw upon this notion throughout to illustrate the potential relevance of these ideas to pathology.

A prominent, but controversial, hypothesis in research on neurodegenerative conditions is that deficits in acetylcholine underwrite the mnemonic deficits characteristic of Alzheimer's disease (Perry, 1986; Contestabile, 2011). This has sometimes been extended to other neurodegenerative conditions (Terry and Buccafusco, 2003), and to normal aging (Bartus et al., 1982). It is important to emphasize that the theory and simulations in this paper are not intended to address the cholinergic hypothesis in neurodegeneration. While we appeal to computational theories concerning the role of acetylcholine in health and hallucinatory disorders, this work should not be seen as supporting (or refuting) the cholinergic hypothesis of cognitive decline in dementia: this work just offers a computational (i.e., teleological) formulation of cholinergic neurotransmission that may help understand psychopathology.

## Active inference

Under active inference, creatures minimize their free energy through action and perception (Friston et al., 2010). Free energy is a functional of beliefs about the processes causing sensations, and a function of sensory data. It is an upper bound on the negative evidence for these beliefs (Beal, 2003), meaning minimisation of free energy is formally equivalent to maximization of evidence for a model of the world (Hohwy, 2016). This (generative) model expresses beliefs about how sensory data are generated, and can take subtly different forms, depending on the type of inference being performed (Friston et al., 2017b). In this paper, we describe inference using a Markov Decision Process (MDP) model:

$$\begin{array}{l} P\left(õ,\tilde{s},\pi ,\zeta \right)=\underset{priors}{\underset{︸}{P\left(\pi \right)P\left(s_{1}\right)P\left(\zeta \right)}}\underset{\tau }{\prod }\underset{transitions}{\underset{︸}{P\left(s_{\tau +1}|s_{\tau },\pi \right)}}\underset{likelihood}{\underset{︸}{P\left(o_{\tau }|s_{\tau },\zeta \right)}} \end{array}$$

Here, $õ={\left[o_{1},o_{2},\ldots ,o_{T}\right]}^{T}$ is a sequence of sensory observations over time. $\tilde{s}$ is the sequence of latent (hidden) states that are not directly observable. π is the policy (or sequence of actions) the subject pursues. The equation above says that observations depend only upon the latent states at the current time step and a precision term, ζ (Parr and Friston, 2017c). Intuitively, if ζ = ∞ this probability (i.e., likelihood) becomes deterministic. Conversely, if ζ = 0 the likelihood of any observation is completely random; irrespective of the state of the world.

For people who are not familiar with the notion of precision, precision quantifies the inverse variability or uncertainty associated with a probability distribution. For example, if the probability of an observed consequence, given a particular cause is very precise, then one can be confident that the observed outcome can be attributed to a particular cause. Conversely, if contingencies are imprecise, there is no definitive relationship between causes and consequences and observations do little to resolve uncertainty about causes. In this sense, precision corresponds to the confidence with which one can infer a cause from observations or data. If one believes contingencies are very precise, one will afford greater weight to sensory evidence in terms of updating beliefs about their causes. This is why optimizing the precision *per se* has been associated with attention. Clearly, getting precision wrong can have profound effects on inference; particularly, in hierarchical inference where the relative precision at different levels of an inference hierarchy becomes especially important—and sometimes counterintuitive (as we will see below). Heuristically, getting precision wrong can lead to both false positives and false negatives, which—in the context of psychopathology—may be the formal homologue of hallucinosis and (attentional) neglect.

States depend upon the states at the previous time step, and the policy currently being pursued. Technically, these probabilistic dependencies are called empirical priors. In addition to the likelihood and empirical priors, three (full) prior distributions are needed: these are the prior over policies, the initial state, and the precision. Active inference mandates that the first of these has a very specific form. However, before we specify this form, we have to consider the form of the (approximate) posterior beliefs, *Q*, an MDP agent holds about the latent variables. To make inference tractable, we employ a mean-field approximation (Friston and Buzsáki, 2016) that assumes the beliefs may be expressed as a product of (marginal) factors:

$$\begin{array}{l} Q\left(\tilde{s},\pi ,\zeta \right)=Q\left(\pi \right)Q\left(\zeta \right)\underset{\tau }{\prod }Q\left(s_{\tau }|\pi \right) \end{array}$$

These are the beliefs that are optimized to minimize free energy, where the approximation allows us to optimize each factor independently. We can now write the free energy under a given policy as

$$\begin{array}{l} F\left(\pi \right)=E_{Q}\left[\ln\;Q\left(\tilde{s},\zeta |\pi \right)-\ln\;P\left(õ,\tilde{s},\zeta |\pi \right)\right] \end{array}$$

The notation *E*_*Q*_[ ] means the expectation (or average) under the approximate posterior beliefs. For actions to minimize this quantity, policies should be selected that lead to lower free energies. However, the free energy is a function of observations, and cannot be defined in relation to observations that have yet to occur. To solve this problem, we can define an expected free energy, and use this to define prior beliefs about policies that minimize this quantity.

$$\begin{array}{lll} G\left(\pi \right) & = & E_{\tilde{Q}}\left[\ln\;Q\left(\tilde{s},\zeta |\pi \right)-\ln\;P\left(õ,\tilde{s},\zeta |\pi \right)\right]\\ \tilde{Q}\left(õ,\tilde{s}|\pi \right) & = & Q\left(\tilde{s}|\pi \right)P\left(õ|\tilde{s}\right)\\ P\left(\pi \right) & = & \sigma \left(-G\left(\pi \right)\right) \end{array}$$

The key move here is to augment the distribution used for the expectation so that it includes beliefs about future observations. This allows us to move from a free energy to an expected free energy. The third line says that those policies considered *a priori* more probable are those that lead to a low expected free energy. Minimizing expected free energy is equivalent to minimizing uncertainty about states of the world. In the absence of any explicit rewards (i.e., prior preferences over outcomes), this uncertainty resolving behavior dominates policy selection—to select policies with the greatest epistemic value or affordance.

This sort of evidence accumulation depends upon the precision afforded sensory evidence, relative to prior beliefs. Crucially, the likelihood or sensory precision **ζ** itself has to be inferred (*via* minimization of free energy). This means that while policies are selected to minimize uncertainty, this selection is based upon posterior expectations about the precision of sensory evidence; namely, expected uncertainty that is determined by the ability of predictions to explain the outcomes encountered.

**Figure 1:**
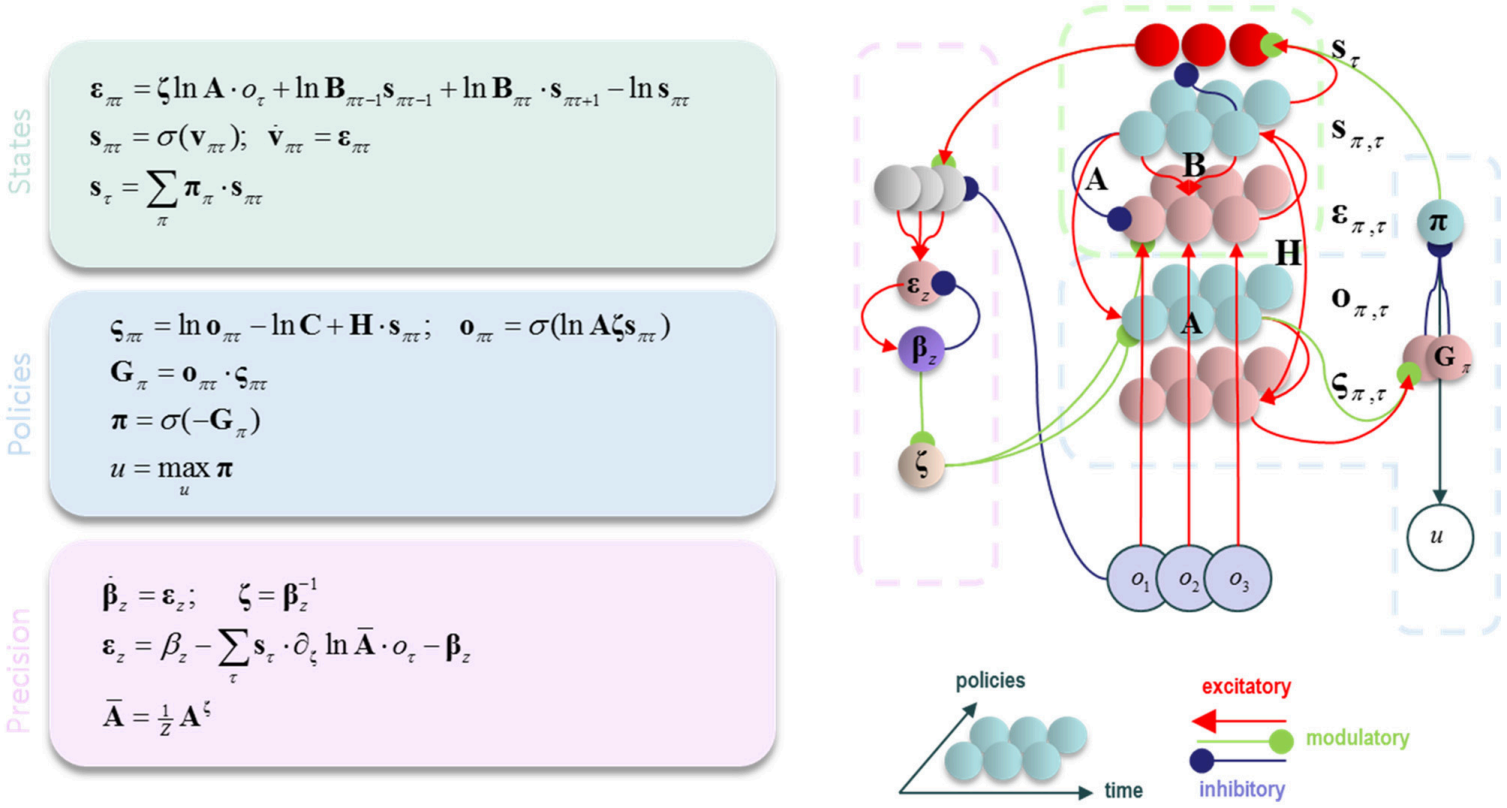
Belief propagation and neuronal (variational) message passing. The equations on the left show the update equations that implement a gradient descent on variational free energy. Please refer to Table 1 and the main text for definitions of the variables used. On the right, the update equations are depicted as a neural network. This resembles the laminar structure of a cortical column, with loops through subcortical structures. The dotted lines highlight the parts of this network that correspond to the equations in the (color-matched) panels. σ is a softmax (normalized exponential) function that ensures the posterior probability distribution sums to one.

Having specified the generative model, we can now compute the free energy gradients for each posterior belief (please see the appendix), and use these to derive the variational message passing equations needed for belief updating over time (Friston et al., 2017a,b; Parr and Friston, 2017c). These are shown in Figure 1 both as belief update equations and graphically as a neural network. There are two important points to draw from this sort of belief update or propagation scheme. First, the structure of the neural network closely resembles that of a cortical column, with loops through subcortical structures (Friston et al., 2017c). Second, *ζ* appears as a multiplicative factor, controlling the gain of messages derived from sensations (Feldman and Friston, 2010). We have previously associated this term with cholinergic projections to the cortex (Parr and Friston, 2017c). This is consistent with empirical data suggesting that acetylcholine mediates the gain of visual cortical responses (Disney et al., 2007), and with the cortical hypometabolism that occurs following lesions to cholinergic forebrain nuclei (Motohiro et al., 1987).

**Table 1:** Glossary of variables (in the text and figures).

**Variable**	**Definition**
**G**_π_	Expected free energy
**π**	Policy posterior
${\text{s}}_{\pi \tau }^{\left(i\right)}$	State posterior belief (for a given policy and time) at hierarchical level *i*
**o**_πτ_	Outcome belief (for a given policy and time)
*o*_τ_	Observed outcome
**A**_*ij*_ = *P*(*o*_τ_ = *i*|*s*_τ_ = *j*, ζ = 1) ${\bar{\text{A}}}_{ij}=P\left(o_{\tau }=i|s_{\tau }=j,\zeta \right)$	Likelihood matrix (mapping states to outcomes)
**B**_*ij*_(*u*) = *P*(*s*_τ+1_ = *i*|*s*_τ_ = *j*, π(τ) = *u*)	Transition matrix (mapping states to states)
**C**_τ*i*_ = *P*(*o*_τ_ = *i*)	Outcome prior
${\text{H}}_{i}=\underset{j}{\sum }P\left(o_{\tau }=j|s_{\tau }=i,\zeta \right)\ln\;P\left(o_{\tau }=j|s_{\tau }=i,\zeta \right)$	Entropy of the likelihood mapping
β_*z*_*P*(ζ) = *Gamma*(1, β_*z*_) **β**_*z*_*Q*(ζ) = *Gamma*(1, **β**_*z*_)	Parameter of prior and posterior beliefs about sensory precision
$\zeta =E_{Q\left(\zeta \right)}\left[\zeta \right]={\text{β}}_{z}^{-1}$	Posterior expectation of sensory precision

## The generative model

**Figure 2:**
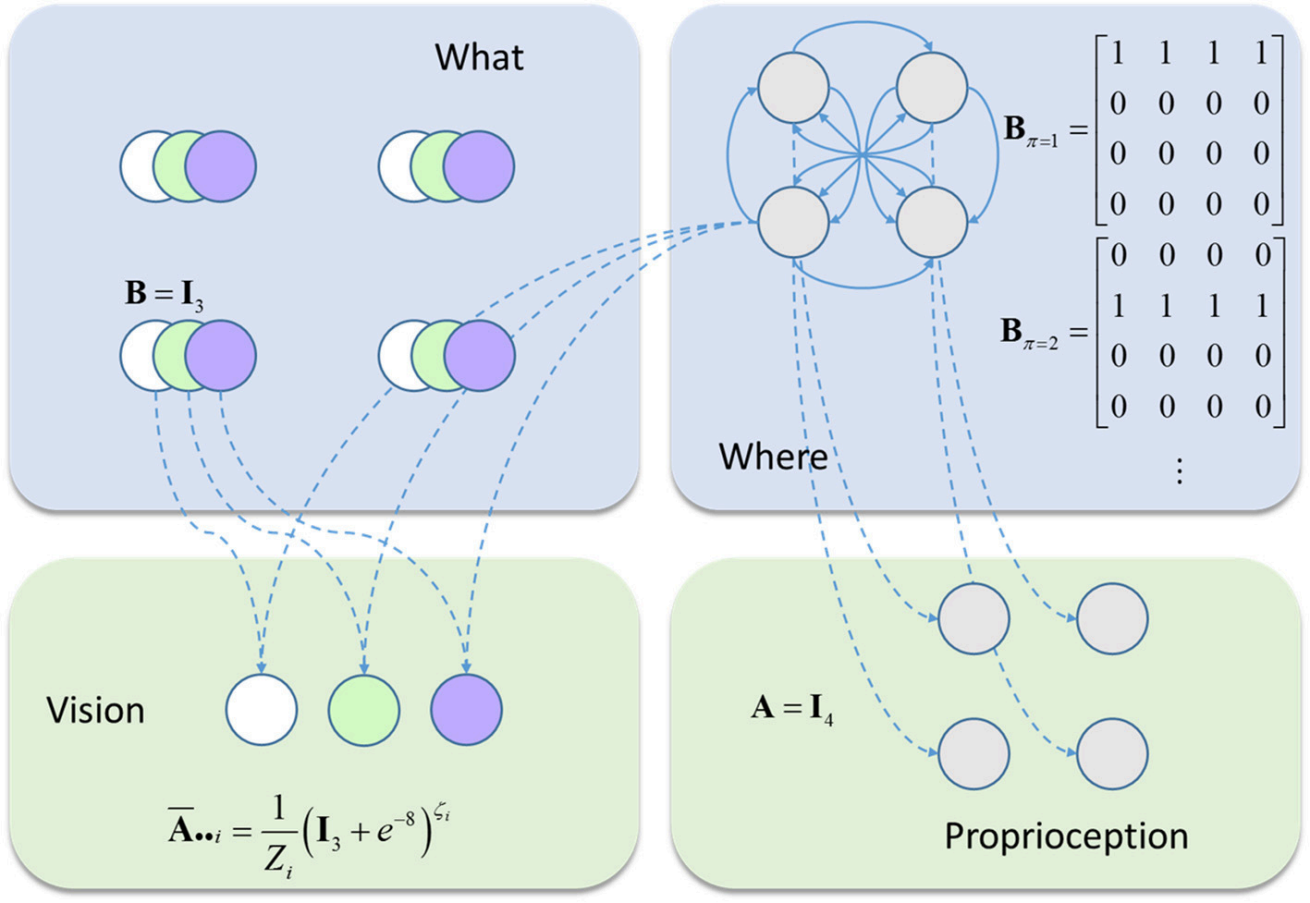
The generative model. This schematic illustrates the form of the generative model used in our simulations. Blue panels show the hidden states: four stimuli at different locations (upper left) and the fixation location (upper right). The latter is a control state (entailed by the policy), with transition probabilities that depend upon policy choices. The green panels show the observable outcomes that are caused by the hidden states. There is one visual and one proprioceptive outcome. The visual outcome depends on the fixation location and what is at that location. The proprioceptive outcome depends only upon the fixation location. The precision of the visual outcome depends upon the current fixation location.

We have outlined above the form of a generic Markov Decision Process, and of the message passing (Dauwels, 2007) it entails. We now focus on a more concrete example. This is the model we will use to illustrate inferences about precision (Figure 2). It comprises a very simple visual scene, containing only four features. Each of these is a circle that may be absent (white), green, or blue. Only one of these features may be foveated at any one time (Mirza et al., 2016). This means that there are four hidden state variables representing visual features, and a fifth that represents fixation location (Parr and Friston, 2017c). The transition probabilities associated with the visual features are all identity matrices, ensuring that the precision of prior beliefs about hidden states is determined only by the prior probability over initial states, *P*(*s*_1_). The transitions between fixation locations depend upon the action (i.e., saccade) selected, and it is this that facilitates the active interrogation of the visual scene (Gibson, 1966).

Two types of sensory data are generated by this model; visual and proprioceptive. The latter is generated by an identity mapping from the fixation location hidden state. Visual data are caused by both the fixation location and the visual feature found at that location. Each location is equipped with its own sensory precision, and beliefs about these precisions can be optimized independently. This generative model should be seen as a toy example that serves to illustrate a subset of pathological changes in visual processing. It is not intended as a complete account of perceptual changes following neurodegenerative insults to the visual system. To demonstrate the behavior of this model, we simulated several different combinations of stimuli and prior belief.

## Simulations

Given this generative model, we can now simulate scene construction via active vision by solving the equations in Figure 1–and examining the simulated behavior and posterior beliefs of a synthetic subject. This approach to simulating visual search behavior has been described in detail in (Mirza et al., 2016; Parr and Friston, 2017c). We will present two sets of simulations. The first uses a simple (single hierarchical level) generative model to illustrate the basics of perceptual inference—and how this depends upon the precision afforded sensory evidence, relative to (empirical) prior beliefs about state transitions. In the second simulation, we equip the model with a second (hierarchical) level that embodies the belief that outcomes are generated by a scene (i.e., a combination of visual objects at four spatial locations) that remains constant over successive (five saccade) visual searches. While this, deliberately simple, form of visual scene does not capture the rich phenomenology associated with real scene construction (Hassabis et al., 2007), it enables us to simulate visual processing under lesions that are hierarchically remote from the (neuromodulatory) effects of expected precision. We offer this as a formal explanation for the sort of (functional) diaschisis that characterizes synucleinopathies; particularly those associated with visual hallucinosis.

**Figure 3:**
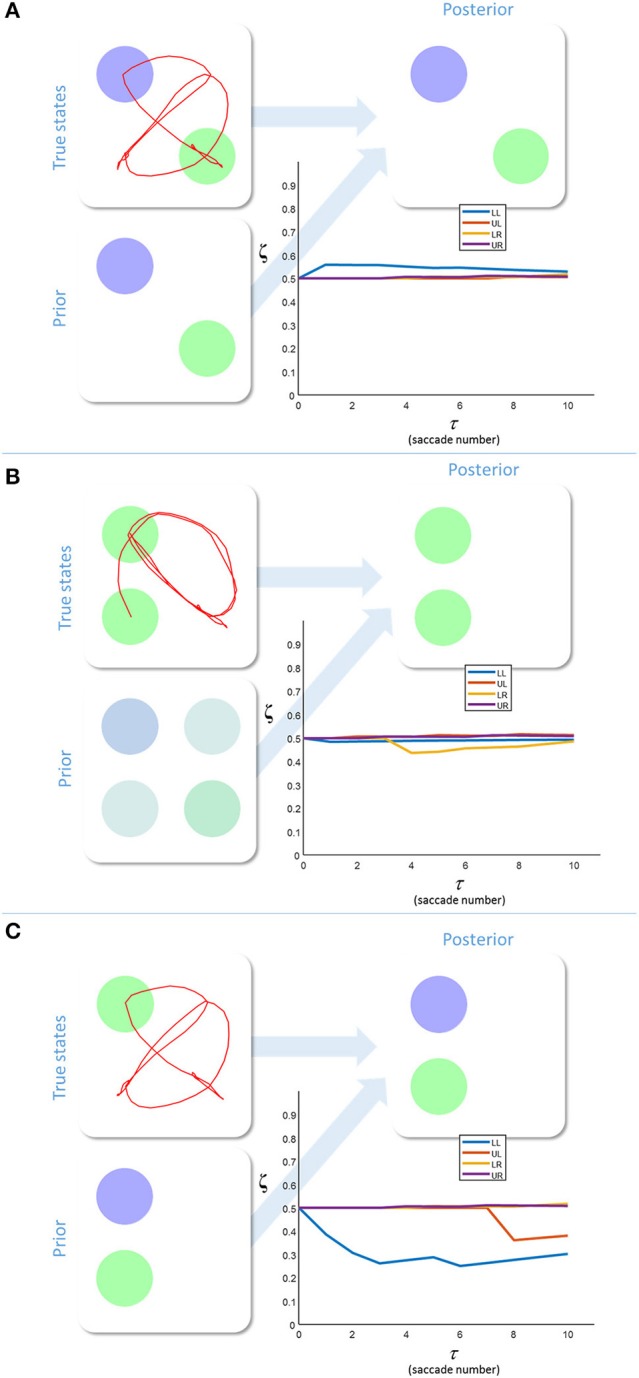
Inferring uncertainty. These three groups of plots show the results of simulating the behavior of a free energy minimizing subject, using the generative model outlined in Figure 2. The prior beliefs about the visual stimuli are depicted by setting the intensity of each color equal to the probability of that color. The posterior beliefs are represented similarly. The true states are presented along with the saccadic trajectory (red line) that determines the sequence in which the stimuli were sampled. The (posterior) sensory precision is shown in the line plots. There is one precision term associated with each location (LL, lower left; LR, lower right; UL, upper left; UR, upper right). **(A)** shows inference with a prior belief that is consistent with the true states. **(B)** shows a relatively imprecise prior that is inconsistent with sensory states. Here, the sensory evidence dominates the inference. **(C)** shows the result of setting a precise prior belief against contradictory sensory data. In this case, the prior dominates, but must induce a decrease in sensory precision in order to do so.

Figure 3 shows the results of simulating a visual search for 10 saccades under different prior beliefs and stimuli. First, we chose a set of prior beliefs that matched the true states of the world (Figure 3A). There is little change in the estimated sensory precision over time, and the posterior belief matches both the prior and the true states. We then tested the case for which the prior and the true states are different. Figure 3B shows a prior belief with the same content as 3a, but held with a lower degree of confidence (i.e., the prior belief is less precise). Again, there is little change in sensory precision, but now the posterior reflects the true states and not the prior. In other words, the sensory likelihood dominates perceptual inference. In Figure 3C, we simulate another mismatch between the prior and sensory evidence. This time, the prior belief is held with a high degree of confidence (i.e., a very precise prior), and this dominates inference. The posterior belief matches the prior, and is inconsistent with the sensory data sampled. The conflict between the prior and the sensory evidence is resolved in this case by a decrease in the precision associated with the contradictory locations. Heuristically, if a prior belief is held very confidently, evidence to the contrary is disregarded or ignored.

We have demonstrated that excessively precise prior beliefs lead to a compensatory decrease in the precision of the likelihood distribution. Given the association between sensory precision and cholinergic signals (Dayan and Yu, 2001; Yu and Dayan, 2002; Vossel et al., 2014; Marshall et al., 2016), this provides a possible mechanism for the decrease in activity in the nucleus basalis of Meynert in several neurodegenerative disorders (Candy et al., 1983). Of these, the synucleinopathies, including Lewy body dementia, show an especially dramatic decrease in cholinergic signaling (Perry et al., 1994), and are associated with false visual inferences (i.e., hallucinations). These are, by definition, the imposition of prior beliefs on perception in the absence of supportive sensory evidence. Furthermore, decreased sensory gain means a smaller response to visual stimulation (Figure 1, first equation), consistent with the combination of reduced occipital cholinergic activity (Kuhl et al., 1996) and occipital hypometabolism (Lobotesis et al., 2001; Heitz et al., 2015) in synuclein disorders.

The above raises an important question: What is the source of the abnormal prior beliefs in conditions such as Lewy body dementia? We have previously argued that pathological prior beliefs might arise through anatomically defined vascular lesions (Parr and Friston, 2017b). Here, too, we can appeal to the anatomical distribution of the lesions to try to understand the relationship between tissue pathology and computational (network level) dysfunction. Lewy body pathology occurs in many brain regions, but it is their presence in parts of the temporal lobe that is associated with visual hallucinations (Harding et al., 2002). This leads us to consider the ventral visual hierarchies and their computational homologs.

## Visual hierarchies

The visual system, like other sensory systems, is known to be hierarchically organized (Desimone et al., 1985; Zeki and Shipp, 1988; Felleman and Van Essen, 1991; Markov et al., 2013). We have previously appealed to this hierarchical structure to model reading (Friston et al., 2017c) and visual working memory tasks (Parr and Friston, 2017d). We now draw upon the same idea to account for the source of the prior beliefs above, and to show how inaccurate but highly precise beliefs can develop. The visual system is organized into two broad hierarchical streams. These are the ventral (what) and the dorsal (where) pathways (Ungerleider and Haxby, 1994). It is the former that is of relevance here, as it leads from the occipital cortex to the temporal cortex, and represents stimulus identity at increasing levels of abstraction. While regions earlier in this pathway tend to respond to simple visual features (Hubel and Wiesel, 1959), later regions are selective for more complex visual objects (Valdez et al., 2015) or scenes (Epstein et al., 1999), constructed from lower level features. This is very important in accounting for the phenomenology of visual hallucinations in neurodegenerative conditions, as hallucinatory components of the percept appear in a consistent and plausible way in the context of the scene. This implies there is no impairment in scene construction *per se*. Instead, it is the wrong scene that is constructed. Crucially, this suggests hallucinated scenes are constructed based upon hierarchical principles, leading to the integration of a false percept in a way that is contextualized by the rest of the scene. This does not imply any impairment in the posterior precision of the overall percept.

**Figure 4:**
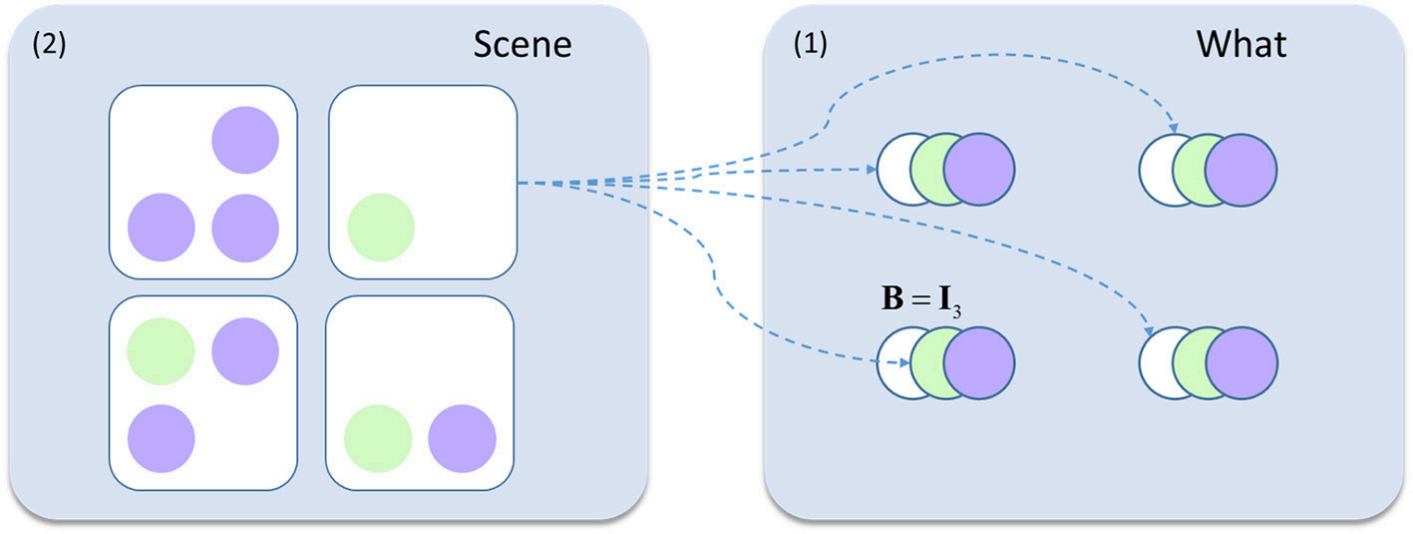
Hierarchical generative model. This schematic shows the addition to the model in Figure 2. The visual scenes represent second level **(2)** causes while the contents of this scene (the ‘what' states from earlier) are the first level **(1)** causes generated by these scenes. For example, the scene containing only a green circle in the lower left quadrant generates white circles in the upper two locations, and a green circle in the lower left. The rest of the generative model is as described in Figure 2.

To account for this hierarchical structure, we can augment our generative model so that visual stimuli (the “what” panel in Figure 2) are themselves generated by “scene” states. Both the “what” and the “scene” variables are types of hidden state. We refer to the former as a “first level” and the latter as a “second level” state. The second level is much simpler in this case (Figure 4), as there is only one type of state with no policies. Furthermore, all transitions at the second level are taken to be identity matrices, expressing the belief that the scene remains constant over time. This type of generative model allows the first level (empirical) priors to be generated by the second level. While this generative model is too abstract to map directly to the real visual system, this type of hierarchy does express cardinal features of the organization of the ventral visual stream.

Importantly, although inference about a visual feature can be performed within a given fixation, it takes a multiple saccades to make inferences about the scenes at the second level. This implies that beliefs at this level should be updated more slowly (Friston et al., 2017c), consistent with the slower response properties higher in sensory hierarchies (Hasson et al., 2008; Kiebel et al., 2008; Murray et al., 2014).

**Figure 5:**
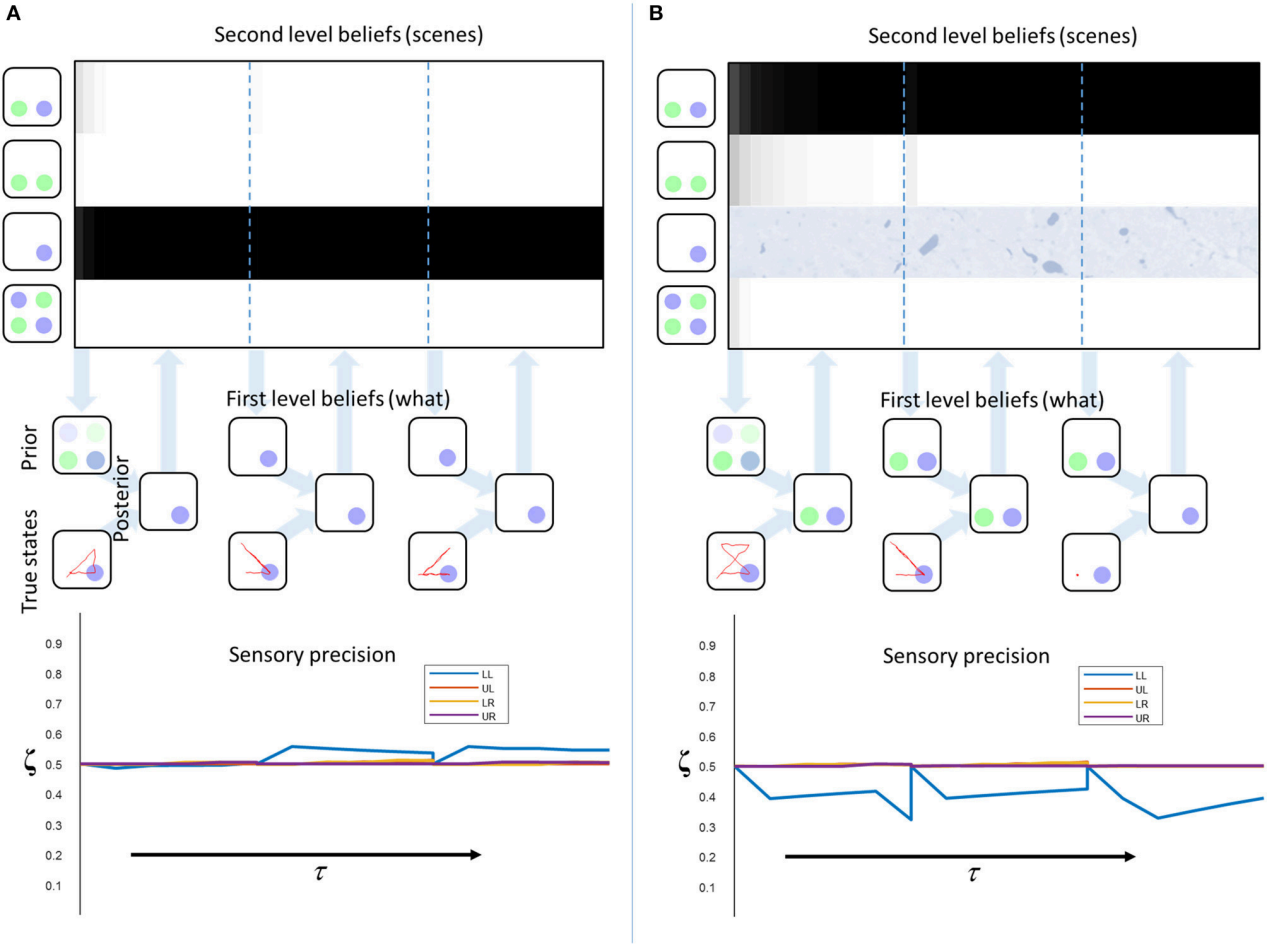
Empirical priors and pathology. These plots illustrate the evolution of beliefs about second level states, first level states, and sensory precision. The upper plots show the beliefs about scenes over time. Each row of these represents a given scene (indicated by the images on the left). The shading indicates the belief that this is the scene responsible for the sensory input. Black indicates a belief that the probability is 1, white indicates 0. The descending arrows represent the computation of a first level empirical prior from the second level beliefs. A new empirical prior is generated after every 5 saccades (demarcated by dashed blue lines). The empirical prior and the sensory consequences of saccadic exploration combine to form first level posterior beliefs (exactly as in Figure 3). The beliefs from each set of 5 fixations are used to update the second level beliefs (ascending arrows). The lower plots show the beliefs about the sensory precision, aligned to the beliefs at the higher level. The precision is reset at the vertical dotted lines. **(A)** shows “healthy” second level priors that associate an equal probability to each scene at τ = 0. Under these priors, the correct scene is inferred and the consistency between priors and sensory data leads to an increase in sensory precision. **(B)** shows the same model but with the prior probability of the third scene set to zero to simulate the loss of this neuron (or neuronal population). Here, the conflict between priors and sensory evidence leads to a decrease in precision. This also demonstrates the importance of action in perception as, at the final time-step, consistently fixating on the lower left location leads to a correct percept. This illustrates the point that collecting more data can compensate for the diminished precision of those data.

Figure 5 shows the inferences made when we simulate responses with this hierarchical model. The upper part of the figure shows the beliefs about each of the second level states through time. At the start, the second level beliefs are combined (weighted by their probabilities) to generate an empirical prior at the first level. In both Figures 5A,B, this prior is relatively imprecise. A sequence of 5 saccades is performed, and the observations made are used to refine the first level posteriors in exactly the same way as in Figure 3. These posterior beliefs are used to update the second level beliefs. These then generate a new empirical prior and this sequence repeats. The sensory precision is reset to its prior value whenever a new empirical prior is set (at the start of each sequence of 5 saccades).

The reason the precision re-sets to its prior value periodically is due to the separation of temporal scales inherent in the generative model. This is analogous to processes like reading, for which a sentence provides a high level context linking sequential words. Letters in one word only inform inferences about the next word via sentence-level representations. This means all lower level representations are set to their (empirical) prior values every time we move from one word to the next. In our setting, the same is true of all lower level representations, including the precision. In a more complex model, it would be possible to condition the prior belief about the precision upon slowly changing variables at the higher level. While beyond the material presented in this paper, this would allow inferences about the precision to transcend the time-scale of the lower level.

Figure 5A illustrates this process in a model with “healthy” second level priors. There is a very rapid inference that the third scene is the most likely cause at the second level, and the first level beliefs, following saccadic interrogation, invariably match the true states. A moderate increase in estimated precision occurs under the second empirical prior, because confident prior beliefs match the sensory inputs. Note that the start location is in the lower left, so there is a larger effect for the precision at this location. This illustrates the fact that the prior has a greater influence at the start of the trial, where fewer observations have been made. Figure 5B shows a simulated synucleinopathy. Neuronal loss (or disconnection) high in the ventral stream has been simulated by setting the second level prior belief for one of the scenes (the correct one) to zero. This is as if we had removed the neuron that represents this second level hypothesis. Interestingly, this does not impede the formation of confident (false) first level empirical priors. As we saw earlier, these induce a decrease in the estimated sensory precision, and false perceptual inference. This demonstrates that pathology high in ventral visual hierarchies can, in principle, induce changes in distant brain areas—something that has been characterized in terms of a functional or dynamic diaschisis (Price et al., 2001; Carrera and Tononi, 2014). The idea that damage to a neuronal population preserves the confidence in the beliefs they represent may seem counterintuitive. However, even with the loss of neurons representing the correct inference, there is still a clear “best” explanation at the level of scenes. This leads to confident posterior beliefs about the scene, giving rise to confident (but incorrect) empirical prior beliefs about the contents of that scene. This is further facilitated by the permissive decrease in sensory precision.

This result recapitulates the idea that, for hallucinations to occur, prior beliefs must be held with a high degree of confidence (precision) relative to that associated with contradictory sensory evidence. This has previously been demonstrated in the context of auditory hallucinations in schizophrenia (Adams et al., 2013). Our account does, however, provide a different perspective on the initial computational insult. While this has previously been formulated as a false prior belief that something is present, we have demonstrated that hallucinations may be induced by a false prior that a given scene is not a good explanation for sensory data. This forces the brain to resort to an alternative explanation, associated with other, spurious, perceptual content.

## Computational neuropathology

In the above, we have presented a model that relates temporal lobe pathology to the development of complex visual hallucinations and reduced cholinergic signaling to the occipital cortex. Crucially, although the primary pathology only affects temporal components of the simulated network, its computational consequences are felt throughout the brain via a dynamic diaschisis (from Greek δ*ιασχισισ* meaning “shocked throughout”). This type of account is necessary in explaining the patterns of diaschisis observed in neuropathological processes.

The synucleinopathies [including Lewy body dementia, Parkinson's disease, and Multiple system atrophy (Tsuboi and Dickson, 2005; McCann et al., 2014)] provide important examples that illustrate the need to connect tissue pathology to computational dysfunction. Despite the presence of physiological changes in the occipital cortices (Kuhl et al., 1996), and visual symptoms (McKeith et al., 2004; Weil et al., 2016), the histopathological processes in these disorders tend not to affect occipital cortex directly (Khundakar et al., 2016). While impaired dopamine signaling to the cortex in these disorders might contribute, occipital regions tend to receive relatively few dopaminergic projections (Javoy-Agid et al., 1989). The absence of these processes in primary visual areas, and the association between visual symptoms and temporal lobe Lewy bodies (Harding et al., 2002), calls for an explanation of physiological changes in the former in terms of their computational relationship to the latter.

We note that, for prior beliefs to dominate inference, the sensory precision must be low relative to the precision of prior beliefs. This means that hallucinations could occur with intact prior beliefs and a primary lesion to systems encoding sensory precision, or an increase in prior confidence without any change in sensory precision. Computationally, these are equivalent as they each change the balance of precisions in the same way. However, they are not necessarily biologically equivalent. The former implies a primary lesion to neuromodulatory systems that modulate synaptic gain in sensory cortices, while the latter implies damage to higher regions of cortex that provide empirical priors to sensory areas. In the context of visual hallucinations in Lewy body disease, both of these are present. While these may be two independent primary lesions, a simpler explanation would be that one is a downstream effect of the other. In this paper, we have suggested a mechanism by which damage to higher cortical areas could lead to disruption of synaptic gain in early visual cortex.

**Figure 6:**
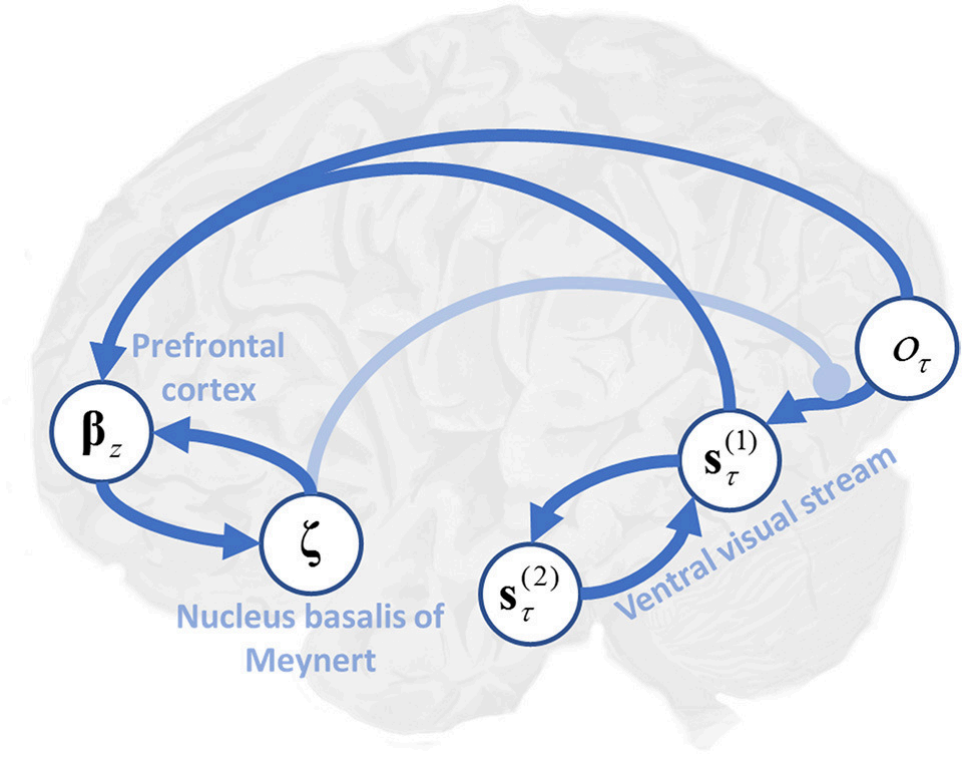
Precision, expected uncertainty and the ventral visual stream. This schematic illustrates the hypothetical computational anatomy of the ventral visual stream and its cholinergic modulation. Visual outcomes (*o*_τ_) are shown in the primary visual cortex. These inform first level beliefs (**s**${}_{\tau }^{\left(1\right)}$) early in the ventral stream, and the connection between these is modulated by cholinergic projections from the basal nucleus in the forebrain. First level beliefs are reciprocally influenced by second level beliefs (**s**${}_{\tau }^{\left(2\right)}$) in the temporal lobe. Here, ($\zeta =E_{Q\left(\zeta \right)}\left[\zeta \right]=\beta_{z}^{-1}$) (see Table 1 and Figure 1 for further details). We have (speculatively) suggested that the prefrontal cortex may be engaged in computing the expected precision, utilizing its inputs from those regions representing first level beliefs, and its connections to the basal forebrain (Zaborszky et al., 1997).

In short, the formal account of active inference or vision on offer here also provides an explanation in terms of a functional diaschisis—a dysfunction of one region as a consequence of a distant lesion (Price et al., 2001; Carrera and Tononi, 2014). Figure 6 illustrates a plausible computational anatomy that could underwrite this account. While this anatomy is speculative, it serves to illustrate the importance of the functional interactions between brain regions to the understanding of neurological disease. Damage to temporal regions, representing second level beliefs, induces changes in the first level beliefs. This leads to inconsistencies between perceptual beliefs and sensory data, which down regulates cholinergic projections to the occipital cortex. Decreased cholinergic signaling uncouples beliefs about states from sensations they cause, facilitating hallucinations (Perry et al., 1991; O'Callaghan et al., 2017). As this model would predict, treatment with cholinesterase inhibitors increases occipital blood flow, while attenuating visual hallucinations (Mori et al., 2006). A complementary, and more biophysically detailed, perspective on this is formulated in terms of impaired conductance (Tsukada et al., 2015) in the synapses between visual cortical neurons, and those in the ventral visual stream.

An influential model of recurrent complex visual hallucinations (Collerton et al., 2005) implicates these same regions, but makes the point that many other disorders involve similar changes. For example, cholinergic deficits are also associated with Alzheimer's disease (Minoshima et al., 2004), although to a lesser extent (Perry et al., 1994; Tiraboschi et al., 2000). Like those for Lewy body disease, pharmacological therapeutics have focused on correcting this neurochemical deficit (Lam et al., 2009). There is evidence to implicate changes in the temporal lobes in this disorder, as it tends to impact these structures early. However, this is typically more medial than in Lewy body dementia (Minoshima et al., 2002). Furthermore, it is unlikely that the cholinergic deficits in Alzheimer's disease are consequences of temporal lobe changes, as there is good evidence for a primary pathological insult to the nucleus basalis (Etienne et al., 1986; Samuel et al., 1994; Liu et al., 2015). This renders it improbable that this condition exhibits a similar set of computational deficits to those described above. The lower prevalence of visual hallucinations in Alzheimer's disease, despite overlapping pathological features with Lewy body disease, illustrates an important point. It is not sufficient to have temporal lobe damage and cholinergic dysfunction to give rise to hallucinations. The interplay between the two is crucial in characterizing this type of diaschisis.

In this paper, we have focused upon false positive inferences (i.e., hallucinations). However, brain damage often leads to false negative inferences (i.e., agnosia) (Warrington and James, 1967). These manifest as a failure to perceive a stimulus, despite it being present. The approach we have described could be used to account for these phenomena in several ways. We outline these here, but emphasize that determining which of these best accounts for agnosia remains an open question that requires further investigation. The first way in which we could account for this is by setting a prior belief that a given object is present to zero. If the most probable alternative explanation is the absence of any object, this inference will result. It is important to distinguish this inference of absence from uncertain inferences, in which the presence or absence of an object cannot be inferred with any certainty. These could result from disconnections that render this object conditionally independent from sensory data in the generative model. This would ensure beliefs about the presence or absence of a given object would not depend upon these data. A third way in which certain stimuli may fail to enter into perceptual awareness is the failure to attend to certain kinds of stimuli, as in visual neglect (Halligan and Marshall, 1998). We have previously argued that this syndrome, in which stimuli on the left of space are ignored, depends upon a failure to actively engage with stimuli on the left (Parr and Friston, 2017b).

A number of outstanding questions are raised by the approach we have taken, which require empirical resolution. The first concerns our use of the term “visual features.” We have illustrated a feature as the color of a circle in a given location, but this is not mandated by the mathematics used in our generative model. In principle, relevant features could be shape, luminance, contrast, or any other experienced attribute. We would need to present patients with a task like that illustrated above, but with different sorts of stimuli, to elucidate which of these afford the right level of description—and whether the ensuing responses are conserved over patients. The second question concerns the fixed parameters of the generative model—such as the prior belief about sensory precision. These are likely to be subject specific, but could be estimated from eye-tracking data collected during the above task (Mirza et al., 2018). Finally, we would hope to use these data to fit the prior beliefs (at the second level) of both patients and healthy participants (i.e., a quantitative and belief based computational phenotyping). We predict that patient data would offer greatest evidence for a reduced (Friston et al., 2016) version of the model that best fits healthy participants. If patient data afforded greater evidence for any other model, this would provide evidence against the hypotheses advanced in this paper. Given the associations between the parameters of our model and their biological substrates, this makes additional, falsifiable, predictions. First, if we were to perform this behavioral experiment combined with neuroimaging, we would predict a reduced effective connectivity between early visual areas and regions in the ventral visual stream that correlates with behaviorally derived precision parameters. Second, chemical neuroimaging to assess cholinergic dysfunction should correlate with both behaviorally derived precision parameters, and decreases in effective connectivity in the visual system. Evidence against these associations would represent evidence against our hypothesis.

## Conclusion

In the first part of this paper, we illustrated the computational mechanisms that could act to maintain the perceptual balance between prior beliefs and sensory evidence. We simulated inferences about the precision associated with the likelihood, and demonstrated that confident, but incorrect, prior beliefs cause a decrease in the expected sensory precision, and false perceptual inferences. In the second part, we asked what the computational mechanisms might be that give rise to pathological empirical priors, and motivated this through an appeal to the neurobiology of synuclein disorders. We described a plausible mechanism by which tissue pathology in higher visual areas could cause in occipital hypometabolism, cholinergic deficits, and visual hallucinations. Crucially, this calls upon the computational (network level) pathologies induced by regional synucleinopathies. This accounts for several empirical findings, including the association of temporal lobe changes with hallucinations in Lewy body disease and the improvement in hallucinations and occipital metabolism when these patients are treated with cholinesterase inhibitors. The ideas and simulations presented here emphasize the importance of relating neuropathological processes to computational dysfunction to understand neurological disease.

## Software note

Although the generative model changes from application to application, the belief updates described in this paper are generic and can be implemented using standard routines (here a customized version of **spm_MDP_VB_X.m**). These routines are available as Matlab code in the SPM academic software: http://www.fil.ion.ucl.ac.uk/spm/. Simulations of the sort reported above can be reproduced (and customized) via a graphical user interface by typing in >> **DEM** and selecting the “visual foraging” demo.

## Author contributions

TP wrote and performed the simulations using an inversion scheme written by KF. TP, DB, PV, and KF contributed to conception and writing of the manuscript.

### Conflict of interest statement

The authors declare that the research was conducted in the absence of any commercial or financial relationships that could be construed as a potential conflict of interest.

## Appendix

In this appendix, we outline the derivations for the update equations we have used in our simulation. For more technical accounts, please see (Friston et al., 2017a,b; Parr and Friston, 2017c).

### Inference

The free energy for a given policy is given as

$$\begin{array}{l} F\left(\pi \right)=E_{Q}\left[\ln\;Q\left(\tilde{s}|\pi \right)-\ln\;P\left(õ,\tilde{s}|\pi ,\zeta \right)\right] \end{array}$$

If we are interested in finding *Q*(*s*_τ_|π), we can omit all terms that are constant with respect to this

$$\begin{array}{l} F(\pi )=E_{Q(s_{\tau }|\pi )}[\ln Q(s_{\tau }|\pi )]-E_{Q(s_{\tau -1}|\pi )Q(s_{\tau }|\pi )}[\ln P(s_{\tau }|s_{\tau -1},\pi )]\\ \;-E_{Q(s_{\tau }|\pi )Q(s_{\tau +1}|\pi )}[\ln P(s_{\tau +1}|s_{\tau },\pi )]\\ \;-E_{Q(s_{\tau }|\pi )}[\ln P(o_{\tau }|s_{\tau },\zeta )] \end{array}$$

In terms of sufficient statistics, this is

$$\begin{array}{l} F_{\pi }=s_{\pi \tau }\cdot (\ln s_{\pi \tau }-\ln B_{\pi \tau -1}s_{\pi \tau -1}-\ln B_{\pi \tau }\cdot s_{\pi \tau +1}\\ \;\;-\zeta \ln A\cdot o_{\tau }) \end{array}$$

We can then set up a gradient descent on this using an auxiliary variable, **v**_πτ_:

$$\begin{array}{lll} {\text{s}}_{\pi \tau } & = & \sigma \left({\text{v}}_{\pi \tau }\right)\\ {\overset{{}^{\circ}}{\text{v}}}_{\pi \tau } & = & -\nabla_{\text{s}}{\text{F}}_{\pi }={\text{ε}}_{\pi \tau } \end{array}$$

$$\begin{array}{l} {\text{ε}}_{\pi \tau }=\ln\;{\text{B}}_{\pi \tau -1}{\text{s}}_{\pi \tau -1}+\ln\;{\text{B}}_{\pi \tau }\cdot {\text{s}}_{\pi \tau +1}+\text{ζ}\ln\;\text{A}\cdot o_{\tau }-\ln\;{\text{s}}_{\pi \tau } \end{array}$$

Here, *σ* is a softmax (normalized exponential) function.

### Planning

In order to treat planning as inference (Botvinick and Toussaint, 2012), we must define the free energy to include beliefs about policies. This is:

$$\begin{array}{l} \text{F}=\text{π}\cdot \left({\text{F}}_{\pi }+{\text{G}}_{\pi }+\ln\;\text{π}\right) \end{array}$$

If we set the gradient of the free energy to zero, we find:

$$\begin{array}{l} \nabla_{\text{π}}\text{F}=0\Leftrightarrow \text{π}=\sigma \left(-{\text{F}}_{\pi }-{\text{G}}_{\pi }\right) \end{array}$$

### Precision

We can include beliefs about the precision of the likelihood by defining $\bar{\text{A}}=\frac{1}{Z}{\text{A}}^{\zeta }$. We then express beliefs about ζ as gamma distributions (for which $\text{ζ}=E_{Q}\left[\zeta \right]={\text{β}}_{z}^{-1}$), and write down the free energy as:

$$\begin{array}{l} F=-\pi \cdot \sum_{\tau }s_{\pi \tau }\cdot (\ln\bar{A}\cdot o_{\tau }+\ln B_{\pi \tau }\cdot s_{\pi \tau +1})+\ln{\text{β}}_{z}\\ \;-\ln\beta_{z}+\zeta \beta_{z} \end{array}$$

$$\nabla_{\zeta }F=0\Leftrightarrow_{z}=\beta_{z}-\sum_{\tau }s_{\tau }\cdot \nabla_{\zeta }\ln\bar{A}\cdot o_{\tau }$$

$${\dot{\text{β}}}_{z}=\beta_{z}-\sum_{\tau }s_{\tau }\cdot \nabla_{\zeta }\ln\bar{A}\cdot o_{\tau }-{\text{β}}_{z}$$
